# Factors associated with parents’ hesitancy to vaccinate their children against COVID-19: The moderator role of parental anxiety

**DOI:** 10.1177/13591053251323502

**Published:** 2025-03-17

**Authors:** Josée Richard, Anik Dubé, Jalila Jbilou, Mylène Lachance-Grzela

**Affiliations:** University of Moncton, Canada

**Keywords:** access to information, adolescents, children, choice overload, COVID-19 vaccine hesitancy, mistrust toward authorities, parental anxiety, perceived freedom of choice

## Abstract

The aim of this study was to examine the factors that influenced parental hesitancy toward vaccinating children against COVID-19 in the months leading up to the launch of the pediatric vaccination campaign. We examined whether parental anxiety moderated the relationships between parents’ access to vaccine information, choice overload, perceived freedom of choice, mistrust toward authorities, and hesitancy toward vaccinating children against COVID-19. A sample of 440 Canadian parents of children aged 1–16 years completed questionnaires. Results revealed that having less access to information and perceiving greater freedom in decision-making increased hesitancy among parents, especially when they reported experiencing anxiety in their parental role. Mistrust of authorities and choice overload were linked to greater hesitancy about vaccination. However, these links were not moderated by the reported parental anxiety. Considering that there will likely be more pandemics in the future, our study has pertinent implications for the healthcare community.

Within the scientific community, there is consensus that vaccination is simple, safe, and one of the most effective preventative measures for protecting us against the ravages of disease and infection. Vaccination has contributed to a substantial reduction in the burden of vaccine-preventable diseases (Kroger et al., 2023; Suryawanshi and Biswas, 2023). Early in the COVID-19 pandemic, many scientists and policy makers were concerned about vaccination uptake among the general population (Roozenbeek et al., 2020). Regarding the effectiveness of a COVID-19 vaccine, it was estimated that achieving herd immunity would require vaccinating at least 75% of the eligible population, and ideally even more (Vyas et al., 2020). However, vaccination hesitancy, meaning either delaying or refusing to get vaccinated or to get one’s children vaccinated, is quite common (Roozenbeek et al., 2020). Early estimates suggested that one in seven Canadians could refuse or wait to be vaccinated against COVID-19 (Statistics Canada, 2020). At the time, researchers argued that vaccine hesitancy represented one of the most significant barriers to the ability to control the spread of COVID-19 and reduce transmission rates and economic impact (Roozenbeek et al., 2020). Early in the pandemic, little was known about Canadian parents’ intention to vaccinate their children against COVID-19 if Health Canada were to recommend such vaccination. So, considering that it was well documented that many children were not vaccinated on schedule because of a conscious parental choice (Schellenberg and Crizzle, 2020), it appeared important to examine parental hesitancy toward a pediatric COVID-19 vaccine, prompting the development of this study.

The theory of planned behavior (Ajzen, 1991) and the dual-process theory (Agarwal and Malhotra, 2005) were used as the basic theoretical frameworks of this study to investigate the cognitive, social, and psychological factors behind parents’ hesitancy to vaccinate their children. According to the theory of planned behavior, intentions to conduct a behavior are shaped by attitudes, subjective norms, and perceived behavioral control. The dual-process theory (Agarwal and Malhotra, 2005) suggests that decision-making relies on both cognitive (deliberate and analytical) and affective (automatic and intuitive) information processing. Factors influencing attitudes (e.g. mistrust toward authorities), perceived behavioral control or cognitive information processing (e.g. lack of access to relevant information) have previously been documented as linked to parental vaccine hesitancy (Damnjanovic et al., 2018; Dubé et al., 2017; Honora et al., 2022; Smith et al., 2017). However, affective factors, such as parental anxiety, are underexplored. The aim of this study was to fill this gap in the literature by examining whether parental anxiety influenced parents’ level of hesitancy regarding the possible vaccination of their children against COVID-19 and whether this anxiety accentuated the impact of known factors contributing to vaccine hesitancy.

## Pediatric vaccination in Canada: An overview

According to the World Health Organization (WHO), incomplete vaccination pertains to children who have missed scheduled vaccinations and thus are partially and/or incorrectly immunized. A recent systematic review by Schellenberg and Crizzle (2020) revealed that the vaccination status for Canadian 2-year-olds, which is the proportion of vaccines received compared to the regulatory compliance, ranged from 50% to 71%. Schellenberg and Crizzle also noted that some children were incompletely vaccinated, meaning they had not received one or more recommended vaccines or all the recommended doses for one or more vaccines. The majority of preschool children in Canada receive vaccinations, but the number of those who had not received any vaccinations by 2 years of age increased from 1.5% in 2013 to 2.3% in 2017 (Health Canada, 2019; Public Health Agency of Canada, 2016). In 2021, although the percentage of completely unimmunized 2-year-old children remained at 2%, the national goal of 95% vaccine coverage for children of this age was not met for any vaccines (Government of Canada, 2021). In Canada, recent data revealed that many 2-year-old children have an incomplete vaccination status (Government of Canada, 2021). More precisely, in 2021, vaccination rates ranged from a high of 91.8% for at least three doses of a vaccine against polio, to a low of 77.1% for four doses of the vaccine against diphtheria, pertussis, and tetanus (DTaP). This data suggested that a significant proportion of parents would likely choose not to vaccinate their children against COVID-19 if such a vaccine was to be approved for pediatric administration.

## Vaccine hesitancy

Vaccine hesitancy is defined as a delay in acceptance or refusal of vaccines despite their availability (MacDonald and SAGE Working Group on Vaccine Hesitancy, 2015). Parental hesitancy has a significant impact on the transmission of vaccine-preventable infectious diseases among children whose parents refuse to get them vaccinated, therefore increasing transmission rates across populations (Siddiqui et al., 2013). Delaying or refusing vaccines can also disrupt herd immunity and create mistrust in the public healthcare system responsible for protecting population health (Damnjanovic et al., 2018; Suryawanshi and Biswas, 2023). Even though the World Health Organization (2019) has designated vaccine hesitancy as one of the 10 leading threats to global health, there is limited data on this issue in the United States (Kempe et al., 2020) and arguably even less in Canada and elsewhere. The decrease in immunization rates for certain vaccines in recent years could be evidence of vaccine hesitancy (Government of Canada, 2021). In a 2016 study by Dubé et al., 40% of parents reported that they experienced hesitation before vaccinating their children. Among them, 58% eventually accepted all the vaccines, 39% accepted some vaccines, and 2% refused all vaccines. On the other hand, 95% of children whose parents were not hesitant were fully vaccinated (Dubé et al., 2016). It was unclear how these data would translate into the COVID-19 context. According to a study by the Government of Canada (2021), 6% of parents of 2-year-old children and 20% of parents of 14-year-old children reported being initially hesitant about vaccinating their child with a vaccine other than influenza, but eventually going ahead with the vaccination. A recent systematic review regarding the COVID-19 vaccination specifically found that vaccine hesitancy was 42.3% for the general Canadian population (Cénat et al., 2022). Hesitancy levels were higher in females than in males, as well as in rural areas. One study found that individuals with children under the age of 18 were more likely than others to report vaccine hesitancy for themselves (Lavoie et al., 2022). The most recent data available reveal that a total of 81% of the overall Canadian population and 67% of children 6 months to 17 years of age have received at least one dose of a COVID-19 vaccine by now (Government of Canada, 2023, 2024). Understanding the vaccine hesitancy experienced by parents prior to the roll-out of a pediatric COVID-19 vaccination could provide insight for future vaccination campaigns.

### Documented correlates of vaccine hesitancy

#### Sociodemographic characteristics

Many sociodemographic factors, including socioeconomic status and education level, have been shown to play a role in the level of hesitation parents experience regarding vaccination. In terms of socioeconomic status, research indicates that predictors of vaccine refusal can differ according to household income. For instance, a study by Carpiano et al. (2019) found that, in Canada, the number of children who did not receive the MMR vaccines was predicted by concerns about side effects in middle to high income households ($60,000–$119,999 CAD) and by their perceived unimportance in low to middle income households ($40,000–$79,999 CAD). Regarding parental education level and its association with the vaccination of children, no obvious patterns have been observed. Kempe et al. (2020) reported that education levels below a bachelor’s degree predicted hesitancy about routine and influenza vaccines. Carpiano et al. (2019) argued that socioeconomic status can influence differences in knowledge, attitudes, and beliefs which mostly center on vaccine side effects and safety concerns. This study also found that lower education and income levels were associated with higher odds of being concerned about vaccines. In sum, many sociodemographic variables have been found to potentially impact a parent’s decision regarding their child’s vaccinations.

#### Access to trusted information and information overload

Having access to trusted information about vaccines, as well as having access to too much information, are both been found to be related to vaccine hesitancy. A systematic review by Smith et al. (2017) showed that having increased information about vaccines was associated with vaccination uptake. In another study conducted among Canadian parents, those who frequently searched for vaccine information and believed it was their parental duty to question vaccines were less inclined to strongly intend to vaccinate their children in the future (Dubé et al., 2017). A study by Kuan (2022) suggested that insufficient vaccine information and a failure to recognize the importance of readily available vaccine information can contribute to parental anxiety regarding their decision to vaccinate their children. However, research also suggests that too much information can create a feeling of information overload and complexify decision-making (Honora et al., 2022). A recent study (Honora et al., 2022) revealed that information overload related to the COVID-19 vaccine resulted in increased skepticism about the vaccine through cyberchondria (anxiety related to excessive searches for medical information on the internet) and a greater perceived risk of the vaccine, which therefore reduced the intention to vaccinate.

#### Mistrust toward authorities

Researchers have found that individuals who express mistrust toward authorities are more reluctant to rely on official sources of information (Damnjanovic et al., 2018; Hudson and Montelpare, 2021). Trusting health authorities and professionals, pharmaceutical companies, law makers as well as a general trust in science has been shown to play an important role in decisions relating to vaccination (Damnjanovic et al., 2018; Dubé et al., 2017; Kuan, 2022; Schellenberg and Crizzle, 2020; Sun et al., 2021; Temsah et al., 2021). A study by Dubé et al. (2017) also found that parents who trusted the information they were given about vaccines were significantly less likely to be hesitant and more likely to eventually accept vaccines, even if they were initially hesitant. Parents with a positive view of the government are more likely to support vaccine policies and perceive them as beneficial rather than restrictive of personal freedom (Damnjanovic et al., 2018). Another recent study revealed that the mistrust of pharmaceutical companies was far more common among non-vaccinating parents (80%) than among those who vaccinate (51%; Greenberg et al., 2017). Overall, the impact of trust on a parent’s intention to vaccinate is an essential consideration.

#### Perceived freedom of choice and choice overload

The perception of freedom while making a decision can have a significant impact on decision-making, similar to the manner in which norms and individuals’ perception of these norms can be influential (Damnjanovic et al., 2018). Perceived freedom has been linked to choice overload in decision-making processes (Damnjanovic et al., 2018; Lau et al., 2015). Decisions and the factors influencing them, can be both exhausting and overwhelming. Often, individuals find it difficult to retain all the necessary information while making decisions (Damnjanovic et al., 2018). A study by Lau et al. (2015) found that the more effort was required to make a decision, the less a person was inclined to feel they had the freedom to make the decision that felt right for them.

In addition, feeling that there are too many confusing and conflicting options can contribute to a lower sense of perceived freedom (Lau et al., 2015). In other words, choice overload can be defined as finding a decision difficult to make due to the large number of options available (Lau et al., 2015). In sum, researchers have found that experiencing choice overload in decision-making reduces an individual’s subjective sense of freedom.

## Parental anxiety

Parental anxiety can involve excessive and intense worrying about one’s child, their happiness, and their eventual success in life (Shirani et al., 2011; Strang, 2015). The pressure parents feel about raising their children can influence their parental decisions (Segrin et al., 2013a; Strang, 2015). Research suggests that anxious parents can favor overprotective parenting behaviors (Strang, 2015), and anxiety is often known to be accompanied by avoidance behaviors. Thomasgard (1998) found that this type of anxiety is associated with a parent who sees their child as vulnerable and considers that overprotective behaviors are the right solution (Thomasgard, 1998; Segrin et al., 2013b). In addition to vaccine hesitancy, parental anxiety seems to be more and more frequent among parents (Strang, 2015).

Given that parental anxiety refers to the excessive worry that parents experience about their children’s well-being, it is possible to assume that it could be linked to vaccine hesitancy (Kuan, 2022; Sun et al., 2021). Parents are often hesitant about vaccinating their children for reasons such as having to witness their discomfort or the possibility of negative side effects (Damnjanovic et al., 2018). They can also experience ambivalence because not vaccinating their children could leave them more vulnerable to contract a preventable infectious disease (Damnjanovic et al., 2018). Often, parents have to deal with social pressures from healthcare professionals, or they feel like they must become vaccination experts to make the best decision for their child (Damnjanovic et al., 2018). In a recent study, many parents stated that their decision not to vaccinate their children was not easily made, and that attempting to interpret the risks and benefits from various information sources often led to uncertainty, confusion, and anxiety (Ward et al., 2018). In sum, being anxious in one’s parenting role when having to navigate the potential risk factors and positive consequences of vaccinating a child could complexify the decision-making process and create a particularly heavy burden for parents. Therefore, it is important to examine parental anxiety and its consequences on parents’ decision-making processes and vaccination decisions.

## The current study

The main goal of this research was to examine the associations between access to information about vaccination, choice overload, perceived freedom of choice, mistrust toward authorities, and parents’ hesitancy about a pediatric COVID-19 vaccination, while also considering the role of parental anxiety. We predicted that insufficient access to information about the vaccine (Hypothesis 1); choice overload (Hypothesis 2); low perceived freedom of choice (Hypothesis 3); and mistrust toward authorities (Hypothesis 4) would be associated to higher levels of COVID-19 vaccine hesitancy among parents, and that the associations would be stronger among parents who experienced heightened levels of anxiety.

A better understanding of the moderator role of parental anxiety in the relationship between these factors and COVID-19 vaccine hesitancy could be pivotal in developing corrective solutions, notably to identify key strategies for future immunization promotion and educational campaigns.

## Method

### Sample

The sample for this study was composed of 440 parents with children between the ages of 1 and 16 years of age. The participants lived in New Brunswick, including 29% (*n* = 126) in rural regions and 66% (*n* = 289) in urban regions (5% did not report their region of residence). Participants were on average 41 years old (SD = 5.22), ranging from 23 to 57 years old. A total of 374 participants (85%) identified as women and 65 as men (15%; none of the participants selected the option “other” for gender). As for the level of education, on average the participants had completed 16.76 years of education (from the first year of elementary to the end of their post-secondary studies, inclusively), which corresponds to an average of a bachelors’ degree. Most participants reported being married (67%, *n* = 293) or living in cohabitation without being married (15%, *n* = 65), whereas 18% (*n* = 82) were divorced or single parents. Participants had an average number of 1.75 children (SD = 0.73). The average annual family income ranged between 100,000$ and 119,000$ CAD. The vast majority of participants (94%) reported that, aside from the annual flu vaccine, their children had received all vaccines recommended by Public Health. Additionally, 5% indicated that their children had received some but not all recommended vaccines, while 1% reported that their children had not received any vaccines.

### Procedure

After approval from the researchers’ institution Research Ethics Board, the recruitment of New Brunswick parents began through emails sent directly to parents via a school district and posts on social media. Interested parents were invited to complete an online survey. Participants were required to read and agree with the information detailed in the consent form before completing the questionnaire. Participants were offered a chance to win one of five gift cards (value of 75$). Data collection occurred in March and April 2021. A quarter of the participants (25.1%) reported having already received a first dose of COVID-19 vaccine at the time of data collection. For context, in New Brunswick, the first COVID-19 vaccine was approved for adults in December 2020 and was made available shortly thereafter to priority groups, including long-term care staff and residents, as well as health care workers. In March and April 2021, priority groups included individuals with complex medical conditions, high school staff, and rotational workers. Eligibility was expanded to all individuals aged 12 and older in late May 2021, to children aged 5–11 in November 2021, and to children aged 6 months to 5 years in July 2022 (Government of New Brunswick, 2024).

### Measures

#### Sociodemographic and routine vaccination uptake questionnaire

The sociodemographic questionnaire consisted of questions relating to age, gender, region of residence, annual family income, level of education, employment, marital status, family structure as well as number and ages of children. Parents were also asked about their children’s routine vaccinations.

#### Pediatric COVID-19 vaccine hesitancy

An adapted version of the Vaccine Hesitancy Scale (Larson et al., 2015; MacDonald and SAGE Working Group on Vaccine Hesitancy, 2015) was used to evaluate participants’ hesitation regarding a future anti-COVID pediatric vaccination. The 10 items of the original scale were adapted to apply to a future anti-COVID vaccine for children. They were answered on a 5-point Likert-type scale ranging from 1 “Strongly disagree” to 5 “Strongly agree”. Sample items are “The COVID-19 vaccine will be important for my child’s health”, as well as “I am concerned the COVID-19 vaccine will have serious adverse effects.” The mean score of the 10 items was calculated after reversing the appropriate items. Higher scores indicate greater hesitancy to a future vaccine against COVID-19 for children. The Cronbach’s alpha was 0.97.

#### Parental anxiety

Parental anxiety was measured with an adapted version of the Anxiety in Parental Role subscale from the EMBU (Swedish acronym for “My memories of upbringing”; Grüner et al., 1999). The 10-item original subscale, which was created to evaluate a child’s perception of their parents’ level of anxiety in their parental role, was modified to be completed by the parents themselves. Examples of the adapted items are: “I am scared by the idea that something could happen to my child” and “I worry about what my child does after school”. The questions were answered on a 4-point Likert scale varying from 1 “Never” to 4 “Always”. To calculate the total score on this scale, the mean score of the 10 items was calculated. The higher the score, the more anxiety the parent experiences in their parental role. The Cronbach’s alpha obtained in this study is 0.83.

#### Previously documented correlates of vaccine hesitancy

##### Mistrust toward authorities

A sub-scale adapted by Jolley and Douglas (2014) measured feelings of disillusionment by the participants toward those involved in vaccinations (e.g. the government, pharmaceutical companies). It consists of 4 items measured on a 6-point Likert-type scale ranging from 1 “Strongly disagree” to 6 “Strongly agree”. Sample items are “I feel tricked, cheated, or deceived by those who are involved in vaccination.” and “I am very disappointed with those who are involved in vaccination.” A mean score was calculated, where higher scores indicate that participants have more feelings of mistrust toward authorities. The Cronbach’s alpha for this sub-scale was 0.95.

##### Perceived freedom in vaccination decisions

The Experience of Perceived Freedom Questionnaire (Lau et al., 2015) was used to measure the level of perceived freedom parents have experienced when making past vaccine-related decisions for their child. The measure contains 4 items rated on a 7-point Likert-type scale ranging between 1 “Definitely not” to 7 “Definitely yes.” An example of an item is “When making the decision to vaccinate or not to vaccinate my child, I felt free to make my own decision.” After reversing the fourth item, a mean score was calculated with higher scores representing a higher sense of freedom. The Cronbach’s alpha for this scale was 0.86.

##### Choice Overload scale

The Choice Overload scale from Lau et al. (2015) measures the overabundance of choices in a decision-making context. This measure has three items in which participants must rate their level of agreement with each statement on a 7-point Likert-type scale ranging from 1 “Definitely no” to 7 “Definitely yes.” An example of an item is “When making the decision to vaccinate or not to vaccinate my child, I felt overwhelmed.” A mean score was calculated for the total. Higher scores on this scale indicate that the participant felt more overwhelmed when making decisions regarding the vaccination of their child. The Cronbach’s alpha for this scale was 0.87.

##### Access to relevant information

One item from the General Health Styles survey (Gust et al., 2005) assessed the degree to which parents believed they had access to sufficient information to make an informed decision regarding the vaccination of their children. The item “I have access to all the information I need to make good decisions about the vaccination of my child” is answered on a 7-point Likert scale ranging from 1 “Definitely no” to 7 “Definitely yes.” A high score indicates that the parent feels well-informed about vaccines to make a solid decision.

## Results

### Preliminary analyses

Missing data were replaced by average scores when at least 80% of the items were answered on a measure. Descriptive statistics and Pearson’s analyses of correlations were conducted (see supplementary analyses for results). Education level and annual family income were significantly correlated with all variables of interest and were therefore used as covariates in the main analyses.

### Principal analyses

A single stepwise regression analysis was conducted. In step 1, access to information about vaccines, choice overload, low perceived freedom of choice, and mistrust toward authorities were entered as the independent variables, parental anxiety was entered as the moderator, and vaccine hesitancy was entered as the dependant variable. In step 2, education level and annual family income were added to the model as covariates.^1^ Finally, in step 3, the interaction terms between the independent variables and the moderator variable were added (interactions were computed with centered variables). The regression results are summarized in Table 1.

**Table 1.** Hierarchical multiple regression analyses predicting vaccine hesitancy.

Variables	β	S_r_^2^	Δ*R*^2^	Total *R*^2^
Step 1				0.664***
Access to information	−0.07***	0.03		
Choice overload	0.07*	0.01		
Perceived freedom	−0.01	0.00		
Mistrust toward authorities	0.50***	0.49		
Parental anxiety	−0.10	0.01		
Step 2			0.013**	0.677**
Access to information	−0.07**	0.03		
Choice overload	0.05	0.01		
Perceived freedom	−0.02	0.00		
Mistrust toward authorities	0.48***	0.47		
Parental anxiety	−0.14*	0.01		
Education level	−0.03**	0.02		
Annual family income	−0.02*	0.01		
Step 3			0.018***	0.694***
Access to information	−0.05**	0.02		
Choice overload	0.08**	0.02		
Perceived freedom	−0.02	0.00		
Mistrust toward authorities	0.48***	0.48		
Parental anxiety	−0.14*	0.01		
Education level	−0.03**	0.02		
Annual family income	−0.02	0.01		
Access to information × Parental anxiety	−0.09*	0.01		
Choice overload × Parental anxiety	−0.09	0.01		
Perceived freedom × Parental anxiety	0.14**	0.02		
Mistrust toward authorities × Parental anxiety	−0.03	0.00		

* *p* < 0.05. **p < 0.01. ***p < 0.001.

In step 1, the independent variables and moderator explained 66.4% of COVID-19 vaccine hesitancy variance, *F*(5, 390) = 154.03, *p* < 0.001 (*R*_2*ajusted*_ = 0.66). Access to information about vaccines, *t*(390) = −3.71, *p* < 0.001, choice overload, *t*(390) = 2.38, *p* < 0.05, and mistrust toward authorities, *t*(390) = 19.48, *p* < 0.001, were significant predictors of COVID-19 vaccine hesitancy, but perceived freedom of choice and parental anxiety were not.

In step 2, the addition of education level and annual family income as covariates (see Note 1) explained an additional 1.3% of vaccine hesitancy variance, *F*(7, 388) = 115.9, *p* < 0.01. When these covariates were taken into account, access to information about vaccines, *t*(388) = −3.45, *p* < 0.01, and mistrust toward authorities, *t*(388) = 18.45, *p* < 0.001, remained significant, whereas parental anxiety, *t*(388) = −2.16, *p* < 0.05, became significant. Both education level, *t*(388) = −2.61, *p* < 0.01, and annual income, *t*(388) = −2.06, *p* < 0.05, were also significant predictors of vaccine hesitancy. However, choice overload and perceived freedom of choice were non-significant.

Once interactions were added in step 3, the model explained an additional 1.8% of variance of vaccine hesitancy for a total of 68.2% of explained variance, *F*(11, 384) = 79.31, *p* < 0.001. Access to information about vaccines, *t*(384) = −2.75, *p* < 0.01, choice overload, *t*(384) = 2.87, *p* < 0.01, mistrust toward authorities, *t*(384) = 18.83, *p* < 0.001, parental anxiety, *t*(384) = −2.12, *p* < 0.05, education level, *t*(384) = −2.76, *p* < 0.01, remained significant predictors of vaccine hesitancy. However, both perceived freedom of choice and annual family income were non-significant. The interaction between trust toward authority and parental anxiety was not significant. Similarly, the interaction between choice overload and parental anxiety approached, but did not reach the significance threshold *t*(384) = −1.84, *p* = 0.07. The interaction between access to information about vaccines and parental anxiety was significant, *t*(384) = −2.13, *p* < 0.05, as was the interaction between perceived freedom of choice and parental anxiety, *t*(384) = 2.63, *p* < 0.01.

For the interactions that were significant, simple slop tests were conducted to examine whether the relationships between the IVs and parental vaccine hesitancy were significant when anxiety was high (+1 SD) and when it was low (−1 SD). The conditional effects of access to information at high and low values of parental anxiety revealed that the link between access to information and parental anxiety is significant in both cases; when parental anxiety is high (+1 SD), *t*(384) = −2.49, *p* < 0.05, and when parental anxiety is low (−1 SD), *t*(384) = −2.79, *p* < 0.01. These results indicate that parents’ COVID-19 vaccine hesitancy was lower when they perceived having a greater access to information and this association was stronger among parents who experienced lower levels of parental anxiety.

The conditional effects of perceived freedom of choice at high and low values of parental anxiety revealed that the link between freedom of choice and parental anxiety is significant for both values; when parental anxiety is high (+1 SD), *t*(384) = 2.33, *p* < 0.05, and when parental anxiety is low (−1 SD), *t*(384) = 2.14, *p* < 0.05. Therefore, parents’ COVID-19 vaccine hesitancy was higher when they perceived having more freedom in the decision and this association was stronger among parents who experienced higher levels of parental anxiety. The conditional effects of access to information and of perceived freedom of choice are illustrated in Supplemental Figures 1 and 2 respectively.

## Discussion

The results supported all the study hypotheses, either partially or completely. Hypothesis 1 was completely supported because greater access to information about vaccines was shown to have a significant link with lower levels of vaccine hesitancy, and the link was stronger among parents who reported higher levels of parental anxiety than among those with lower levels of parental anxiety. Hypotheses 2 and 4 were partially supported, as higher levels of choice overload and mistrust toward authorities respectively were found to be associated with increased levels of vaccine hesitancy, but these associations were not influenced by the participants’ level of parental anxiety. Finally, Hypothesis 3 was partially supported because the relation between perceived freedom of choice and vaccine hesitancy was moderated significantly by parental anxiety, but in the opposite direction than what was expected. More precisely, a higher sense of perceived freedom in the vaccination decision seemed to create more hesitancy for parents regarding their future decision to vaccinate their children against COVID-19, especially when they experienced high levels of parental anxiety.

In addition, while parental anxiety was not hypothesized as having an individual effect on vaccine hesitancy, but rather accentuating the effect of other factors, it is interesting to note that it was negatively associated to the dependent variable once the other variables and interactions were considered in the model. This pattern suggests that, in the studied context, the more a parent experienced parental anxiety, the less vaccine hesitancy they were likely to experience. This direct link could possibly be explained by the fact that the more anxiety an individual experiences in their role as a parent, the greater intolerance they have for the uncertainty associated with the risks of their children being exposed to COVID-19. Therefore, such an individual would see the vaccine as a possible way to decrease or avoid that uncertainty. McNeil and Purdon’s (2022) found that people with and without an anxiety disorder did not differ in their vaccine hesitancy levels, but that greater intolerance of uncertainty, a specific component of anxiety, was associated with greater hesitancy in those without anxiety disorders and with less hesitancy in those with anxiety (McNeil and Purdon, 2022).

Our findings demonstrate that parents who strongly perceived that they had all the information they needed to make good decisions about the vaccination of their children experienced lower levels of hesitancy regarding a future pediatric COVID-19 vaccination. These results agree with a systematic review performed by Smith et al. (2017), which suggested that having increased access to information about vaccines was associated with higher levels of vaccine uptake. Parents perceive it to be their responsibility to inform themselves and make the most suitable decisions for their children (Kuan, 2022). Our findings suggest that higher levels of vaccine uptake may be explained by decreased vaccine hesitancy. All in all, the research suggests that the more information parents feel they have, the less hesitant they will be about a new vaccine. Therefore, parents with sufficient information may be more prone to vaccinate their children when a new vaccine becomes available. Our results suggest that when high levels of parental anxiety are added to the equation, the association is heightened and so greater access to information about vaccines could mean even lower levels of hesitancy regarding a future pediatric COVID-19 vaccination.

In addition, less perceived freedom in the decision to vaccinate one’s child was associated with lower levels of vaccine hesitancy, and this association was stronger for parents who experienced higher levels of anxiety. The conditional effect with lower levels of parental anxiety was also significant. More precisely, vaccine hesitancy generally decreased when parents perceived they had less freedom in the choice, and this decrease was more pronounced among parents who reported lower levels of parental anxiety. Contrarily to what was hypothesized, perceiving less freedom in the choice to vaccinate a child (i.e. vaccination was strongly encouraged by authority figures and organizations) decreased vaccine hesitancy for parents. In the case of parents who do experience parental anxiety, having more freedom in decision-making may potentially heighten hesitancy, as it may feel like the decision rests completely on their shoulders. They may also therefore experience more ambivalence.

The variables of choice overload and mistrust toward authorities were significantly and directly associated with vaccine hesitancy, but these associations were not significantly moderated by parental anxiety. The significant direct links partially confirm our hypotheses and support past research. A decision can be considered difficult to make when multiple options are available (Lau et al., 2015), and decision-making can feel overwhelming (Damnjanovic et al., 2018). This phenomenon is documented in many different contexts. In fact, research has shown that being presented with too many options can decrease satisfaction or lead to postponing the decision altogether (Blasheck and Noor, 2020). When people have too many choices, they can feel overwhelmed trying to decipher and analyze all options, which can cause ambivalence and increased vaccine hesitancy.

Mistrust toward authorities alone explained a considerable portion of the variance in vaccine hesitancy. This result concurs with previous research documenting that people who express mistrust toward authority figures are more reluctant to rely on official sources of information (Damnjanovic et al., 2018, Hudson and Montelpare, 2021; McNeil and Purdon, 2022). Considering that most of the information regarding COVID-19 vaccines was transmitted by official authority agencies (i.e. health authorities and government officials), this could have affected certain people’s decision-making about the vaccine, especially if they had trouble trusting these sources from the beginning (Damnjanovic et al., 2018; Dubé et al., 2017; Hudson and Montelpare, 2021; Schellenberg and Crizzle, 2020). Our findings support the work of Dubé et al.’s (2017), who found that parents who trusted information about vaccines were significantly less likely to be hesitant and more likely to eventually accept them, even if they showed an initial reluctance or hesitation. This result is also in line with the findings of Damnjanovic et al.’s (2018) and Hudson and Montelpare (2021), who suggested that parents with a positive view of the government are more likely to support vaccine policies and see them as being beneficial. Similarly, another study revealed that mistrusting pharmaceutical companies was more common among non-vaccinating parents than among those who vaccinate (Greenberg et al., 2017).

In terms of the sociodemographic variables included as covariates in the model, our findings corroborate past studies. Lower education levels among parents were associated with higher levels of vaccine hesitancy. Multiple other studies align with our findings and support the argument that lower education levels are associated with higher levels of vaccine hesitancy (Carpiano et al., 2019; Gilbert et al., 2017; Kempe et al., 2020; Kiely et al. 2018; see also Dummer et al., 2012; Martens et al., 2014; O’Donnell et al., 2017, for contrasting results). The effect of household income was not as clear, as this variable did not reach the significance threshold when all other variables of the model were considered.

### Clinical and social implications

This study could have important clinical and social implications. The factors previously known to have an impact on vaccination seem to have persisted in the context of this new vaccination and may also influence future new vaccinations. This study also advances current knowledge regarding the impact of parental anxiety on some of these factors. This result highlights the importance of clinicians, including family doctors and pediatricians, engaging in informed and shared decision-making with parents who are hesitant to vaccinate their children. Our study also suggests the relevance of clinicians verifying that parents consider they have all the information they feel they need to decide about vaccination for their children. Public health vaccination promotion campaigns, including raising awareness and providing clear information about preventable infectious diseases and their impacts on children’s health, as well as the benefits of vaccination to prevent these infectious diseases are to be designed and evaluated.

This study is relevant in that the sample was taken during a very specific time in the midst of a worldwide pandemic as well as during the roll-out of a newly created vaccine. As experts agree, there will likely be more pandemics in the future (Marani et al., 2021) and our results could be very important from a historical and social point of view (see Hudson and Montelpare, 2021; Liang et al., 2021; McNeil and Purdon, 2022, for other exceptions). Working to lessen vaccine hesitancy in general could help us as a society to be better prepared to address emergency situations where new vaccination strategies are required.

### Study limitations and future research

While this study has provided positive contributions to the field, it has certain limitations that should be taken into consideration. For instance, it is a cross-sectional study, and therefore it is impossible either to conclude that a causal relation exists between the variables or to understand the variations or developments in COVID-19 vaccine hesitancy over time. A longitudinal study could examine how access to information, choice overload, perceived freedom of choice, mistrust toward authorities, and parental anxiety evolve over time, and how these factors affect vaccine hesitancy. Another limitation of the study relates to the data collection measures. The subscale used to measure access to relevant information contained a single item. Also, the parental anxiety subscale was adapted for this study as it was originally intended to be answered from the child’s perspective. The results of this study should also be interpreted with caution as they could be very specific to the time of data collection, in the middle of a worldwide pandemic, and not be representative of vaccine hesitancy in general or even current hesitancy regarding vaccines. In addition, the results should be generalized with caution as the sample lacked variety (i.e. mostly females with post-secondary education, from southeast New Brunswick, with children between the ages of 1 and 16). In addition, the vast majority of parents (94%) reported that their children had received all routine vaccinations recommended by Public Health. This relatively high rate of vaccine uptake suggests that the sample may disproportionately include parents who hold generally favorable attitudes toward vaccination. As a result, the study may lack sufficient representation of vaccine-hesitant or vaccine-refusing parents, limiting its ability to capture diverse attitudes and decision-making processes regarding childhood COVID-19 vaccination. Choice overload and freedom of choice in vaccination decisions relate to previous experience with vaccination of children. The predicted power of these variables might have been different or stronger if they pertained specifically to the COVID-19 vaccination decision. Future studies should also aim to explore whether other known factors related to vaccine hesitancy would be impacted when moderated by parental anxiety, such as concerns about secondary effects of vaccines, parental attitudes toward vaccines in general, knowledge about vaccines, experiences with past vaccinations, or perceived severity of the illness, to name a few examples.

### Conclusion

To conclude, our findings support the idea that the factors previously documented as being linked to vaccine hesitancy also applied to the Covid-19 pandemic and, possibly, to the roll-out of other new vaccines. Moreover, our results offer a nuance to these results by considering an additional factor: parental anxiety. More precisely, we found that having less access to information and sensing more perceived freedom in decision-making was associated with more hesitancy among parents about vaccinating their children against COVID-19, especially when they experienced parental anxiety. Furthermore, we found that feeling more mistrust toward authorities and overwhelmed with choices was linked to a higher level of vaccine hesitancy among parents regarding pediatric vaccination against COVID-19. Considering that there will likely be more pandemics in the future and that parental anxiety has become increasingly frequent in society in recent years, our study could have valuable and pertinent implications for individuals, families, organizations, and the healthcare community.

## Supplemental Material

sj-docx-1-hpq-10.1177_13591053251323502 – Supplemental material for Factors associated with parents’ hesitancy to vaccinate their children against COVID-19: The moderator role of parental anxiety Supplemental material, sj-docx-1-hpq-10.1177_13591053251323502 for Factors associated with parents’ hesitancy to vaccinate their children against COVID-19: The moderator role of parental anxiety by Josée Richard, Anik Dubé, Jalila Jbilou and Mylène Lachance-Grzela in Journal of Health Psychology

## Notes

1. A model was also computed in which gender of the respondent and age were also included as covariates, but neither variable was significant and the pattern of results remained the same. For the sake of parsimony, these variables were not included in the model presented here.

## References

[bibr1-13591053251323502] Agarwal J Malhotra NK (2005) An integrated model of attitude and affect: Theoretical foundation and an empirical investigation. Journal of Business Research 58(4): 483–493.

[bibr2-13591053251323502] Ajzen I (1991) The theory of planned behavior. Organizational Behavior and Human Decision Processes 50(2): 179–211.

[bibr3-13591053251323502] Blasheck TL Noor J (2020) The dark side of variety: An economic model of choice overload. The Yale Undergraduate Research Journal 1(38): 1–19.

[bibr4-13591053251323502] Carpiano R Polonijo A Gilbert N , et al. (2019) Socioeconomic status differences in parental immunization attitudes and child immunization in Canada: Findings from the 2013 Childhood National Immunization Coverage Survey (CNICS). Preventative Medicine 123: 278–287. 10.1016/j.ypmed.2019.03.033 30904601

[bibr5-13591053251323502] Cénat JM Noorishad PG Moshirian Farahi SMM , et al. (2022) Prevalence and factors related to COVID-19 vaccine hesitancy and unwillingness in Canada: A systematic review and meta-analysis. Journal of Medical Virology 95(1): e28156. 10.1002/jmv.28156 PMC9538578 36114154

[bibr6-13591053251323502] Damnjanovic K Graeber J Ilic S , et al. (2018) Parental decision-making on childhood vaccination. Frontiers in Psychology 9: 735. 29951010 10.3389/fpsyg.2018.00735 PMC6008886

[bibr7-13591053251323502] Dubé E Gagnon D Ouakki M , et al. (2017) Measuring vaccine acceptance among Canadian parents: A survey of the Canadian Immunization Research Network. Vaccine 36: 545–552. 29233605 10.1016/j.vaccine.2017.12.005

[bibr8-13591053251323502] Dubé E Gagnon D Zhou Z , et al. (2016) Parental vaccine hesitancy in Quebec (Canada). PLoS Currents 8: 1–10. 10.1371/currents.outbreaks.9e239605f4d320c6ad27ce2aea5aaad2 PMC4801332 27019766

[bibr9-13591053251323502] Dummer TJB Cui Y Strang R , et al. (2012) Immunization completeness of children under two years of age in Nova Scotia, Canada. Canadian Journal of Public Health 103(5): e363–e367. 10.1007/BF03404442 PMC6973882 23617989

[bibr10-13591053251323502] Gilbert N Gilmour H Wilson S , et al. (2017) Determinants of non-vaccination and incomplete vaccination in Canadian toddlers. Human Vaccines and Immunotherapeutics 13(6): 1447–1453. 10.1080/21645515.2016.1277847 PMC5489291 28129028

[bibr11-13591053251323502] Government of Canada (2021) Highlights from the 2021 childhood National Immunization Coverage Survey (cNICS). Available at: https://www.canada.ca/en/public-health/services/immunization-vaccines/vaccination-coverage/2021-highlights-childhood-national-immunization-coverage-survey.html#coverage (accessed 5 January 2025).

[bibr12-13591053251323502] Government of Canada (2023) Childhood COVID-19 Immunization Coverage Survey (CCICS): 2023 results. Available at: https://www.canada.ca/en/public-health/services/immunization-vaccines/vaccination-coverage/childhood-covid-19-immunization-coverage-survey-2022-results.html (accessed 5 January 2025).

[bibr13-13591053251323502] Government of Canada (2024) COVID-19 vaccination: Vaccination coverage. Available at: https://health-infobase.canada.ca/covid-19/vaccination-coverage/ (accessed 5 January 2025).

[bibr14-13591053251323502] Government of New Brunswick (2024) History of vaccine use in New Brunswick. Available at: https://www2.gnb.ca/content/dam/gnb/Departments/h-s/pdf/en/CDC/HealthProfessionals/history-of-vaccine-use-in-new-brunswick.pdf (accessed 5 January 2025).

[bibr15-13591053251323502] Greenberg J Dubé E Driedger M (2017) Vaccine hesitancy: In search of the risk communication comfort zone. PLoS Currents Outbreaks 1: 1–11. 10.1371/currents.outbreaks.0561a011117a1d1f9596e24949e8690b PMC5346025 28357154

[bibr16-13591053251323502] Grüner K Muris P Merckelbach H (1999) The relationship between anxious rearing behaviours and anxiety disorders symptomatology in normal children. Journal of Behavior Therapy and Experimental Psychiatry 30(1): 27–35. 10365863 10.1016/s0005-7916(99)00004-x

[bibr17-13591053251323502] Gust D Kennedy A Shui I , et al. (2005) Parent attitudes toward immunizations and healthcare providers: The role of information. American Journal of Preventative Medicine 29(2): 105–112. 10.1016/j.amepre.2005.04.010 16005806

[bibr18-13591053251323502] Health Canada (2019) Preliminary results from the 2017 childhood National Immunization Coverage Survey (cNICS). Available at: https://www.canada.ca/en/services/health/publications/vaccines-immunization/vaccine-uptake-canadian-children-preliminary-results-2017-childhood-national-immunization-coverage-survey.html (accessed 10 October 2024).

[bibr19-13591053251323502] Honora A Wang KY Chih WH (2022) How does information overload about COVID-19 vaccines influence individuals’ vaccination intentions? The role of cyberchondria, perceived risk, and vaccine skepticism. Computer in Human Behavior 130: 107176. 10.1016/j.chb.2021.107176 PMC8730468 35013641

[bibr20-13591053251323502] Hudson A Montelpare WJ (2021) Predictors of vaccine hesitancy: Implications for covid-19 public health messaging. International Journal of Environmental Research and Public Health 18(8054): 2–14. 10.3390/ijerph18158054 PMC8345367 34360345

[bibr21-13591053251323502] Jolley D Douglas KM (2014) The effects of anti-vaccine conspiracy theories on vaccination intentions. PLoS One 9(2): e89177. 10.1371/journal.pone.0089177 PMC3930676 24586574

[bibr22-13591053251323502] Kempe A Saville AW Albertin C , et al. (2020) Parental hesitancy about routine childhood and influenza vaccinations: A national survey. Pediatrics 146(1): e20193852. 10.1542/peds.2019-3852 PMC7329256 32540985

[bibr23-13591053251323502] Kiely M Boulianne N Talbot D , et al. (2018) Impact of vaccine delays at the 2, 4, 6 and 12 month visits on incomplete vaccination status by 24 months of age in Quebec, Canada. BMC Public Health 18: 1364. 30537969 10.1186/s12889-018-6235-6 PMC6288945

[bibr24-13591053251323502] Kroger A Bahta L Long S , et al. (2023) General best practice guidelines for immunization. Centers for Disease Control and Prevention. Available at: www.cdc.gov/vaccines/hcp/acip-recs/general-recs/downloads/general-recs.pdf (accessed 1 March 2024).

[bibr25-13591053251323502] Kuan CI (2022) Vaccine hesitancy and emerging parental norms: A qualitative study in Taiwan. Foundation for Sociology of Health and Illness 44: 692–709. 10.1111/1467-9566.13446 35174516

[bibr26-13591053251323502] Larson HJ Jarrett C Schulz WS , et al. (2015) Measuring vaccine hesitancy: The development of a survey tool. Vaccine 33: 4165–4175. 25896384 10.1016/j.vaccine.2015.04.037

[bibr27-13591053251323502] Lau S Hiemisch A Baumeister R (2015) The experience of freedom in decisions – Questioning philosophical beliefs in favor of psychological determinants. Consciousness and Cognition 33: 30–46. 25528494 10.1016/j.concog.2014.11.008

[bibr28-13591053251323502] Lavoie K Gosselin-Boucher V Stojanovic J , et al. (2022) Understanding national trends in COVID-19 vaccine hesitancy in Canada: Results from five sequential cross-sectional representative surveys spanning April 2020–March 2021. BMJ Open 12(4): e059411. 10.1136/bmjopen-2021-059411 PMC8983402 35383087

[bibr29-13591053251323502] Liang ST Liang ST Rosen JM (2021) COVID-19: A comparison to the 1918 influenza and how we can defeat it. Postgraduate Medical Journal 97: 273–274. 33563705 10.1136/postgradmedj-2020-139070 PMC8108277

[bibr30-13591053251323502] MacDonald N and SAGE Working Group on Vaccine Hesitancy (2015) Vaccine hesitancy: Definition, scope, and determinants. Vaccine 33: 4161–4164. 25896383 10.1016/j.vaccine.2015.04.036

[bibr31-13591053251323502] McNeil A Purdon C (2022) Anxiety disorders, COVID-19 fear, and vaccine hesitancy. Journal of Anxiety Disorders 90: 102598 35780664 10.1016/j.janxdis.2022.102598 PMC9233868

[bibr32-13591053251323502] Marani M Katul GG Pan WK , et al. (2021) Intensity and frequency of extreme novel epidemics. Proceedings of the National Academy of Sciences of the United States of America 118(35): e2105482118. 10.1073/pnas.2105482118 PMC8536331 34426498

[bibr33-13591053251323502] Martens PJ Chateau DG Burland EMJ , et al. (2014) The effect of neighborhood socioeconomic status on education and health outcomes for children living in social housing. American Journal of Public Health 104(11): 2103–2113. 25211758 10.2105/AJPH.2014.302133 PMC4202984

[bibr34-13591053251323502] O’Donnell S Dube E Tapiero B , et al. (2017) Determinants of under-immunization and cumulative time spent under-immunized in a Quebec cohort. Vaccine 35: 5924–5931. 28882440 10.1016/j.vaccine.2017.08.072

[bibr35-13591053251323502] Public Health Agency of Canada (2016) Vaccine coverage in Canadian children: Highlights from the 2013 childhood National Immunization Coverage Survey. Government of Canada. Available at: https://www.canada.ca/en/public-health/services/publications/healthy-living/vaccine-coverage-canadian-children-highlights-2013-childhood-national-immunization-coverage-survey.html (accessed 5 January 2024).

[bibr36-13591053251323502] Roozenbeek J Schneider CR Dryhurst S , et al. (2020) Susceptibility to misinformation about COVID-19 around the world. Royal Society Open Science 7(10): 201199. 33204475 10.1098/rsos.201199 PMC7657933

[bibr37-13591053251323502] Schellenberg N Crizzle AM (2020) Vaccine hesitancy among parents of preschoolers in Canada: A systematic literature review. Canadian Journal of Public Health 111: 562–584. 32783144 10.17269/s41997-020-00390-7 PMC7438392

[bibr38-13591053251323502] Segrin C Givertz M Swaitkowski P , et al. (2013a) Overparenting is associated with child problems and a critical family environment. Journal of Child and Family Studies 24(2): 470–479.

[bibr39-13591053251323502] Segrin C Woszildo A Givertz M , et al. (2013b) Parent and child traits associated with overparenting. Journal of Social and Clinical Psychology 32(6): 569–595.

[bibr40-13591053251323502] Shirani F Henwood K Coltart C (2011) Meeting the challenges of intensive parenting culture: Gender, risk management, and the moral parent. Sociology 46: 25–40.

[bibr41-13591053251323502] Siddiqui M Salmon DA Omer SB (2013) Epidemiology of vaccine hesitancy in the United States. Human Vaccines and Immunotherapeutics 9(12): 2643–2648. 24247148 10.4161/hv.27243 PMC4162046

[bibr42-13591053251323502] Smith L Amlôt R Weinman J , et al. (2017) A systematic review of factors affecting vaccine uptake in young children. Vaccine 35: 6059–6069. 28974409 10.1016/j.vaccine.2017.09.046

[bibr43-13591053251323502] Statistics Canada (2020) Canadians’ willingness to get a COVID-19 vaccine: Group differences and reasons for vaccine hesitancy. Catalogue no. 45280001.

[bibr44-13591053251323502] Strang KR (2015) The Relationship Between Overparenting, Parenting Style, and Anxiety in Parents of School-Aged Children. ProQuest, The Chicago School of Professional Psychology, Ann Arbor, MI.

[bibr45-13591053251323502] Sun R Wang X Lin L , et al. (2021) The impact of negative emotional reactions on parental vaccine hesitancy after the 2018 vaccine event in China: A cross-sectional survey. Human Vaccines and Immunotherapeutics 17(9): 3042–3051. 33950775 10.1080/21645515.2021.1907149 PMC8381837

[bibr46-13591053251323502] Suryawanshi YN Biswas DA (2023) Herd immunity to fight against COVID-19: A narrative review. Cureus 15(1): e33575. 10.7759/cureus.33575 PMC9909126 36779140

[bibr47-13591053251323502] Temsah MH Alhuzaimi AN Aljamaan F , et al. (2021) Parental attitudes and hesitancy about COVID-19 vs. routine childhood vaccinations: A national survey. Frontiers in Public Health 9: 752323. 34722451 10.3389/fpubh.2021.752323 PMC8548678

[bibr48-13591053251323502] Thomasgard M (1998) Parental perceptions of child vulnerability, overprotection, and parental psychological characteristics. Child Psychiatry and Human Development 28: 223–240. 9628055 10.1023/a:1022631914576

[bibr49-13591053251323502] Vyas J Kadam A Mashry R (2020) The role of herd immunity in control of contagious diseases. International Journal of Research and Review 7(7): 108–119.

[bibr50-13591053251323502] Ward PR Attwell K Meyer SB , et al. (2018) Risk, responsibility, and negative responses: A qualitative study of parental trust in childhood vaccinations. Journal of Risk Research 21(9): 1117–1130.

[bibr51-13591053251323502] World Health Organization (2019) Ten threats to global health in 2019 [Press release]. Available at: https://www.who.int/news-room/spotlight/ten-threats-to-global-health-in-2019 (accessed 5 January 2025).

