# Supplementary material for: Factors associated with parents’ hesitancy to vaccinate their children against COVID-19: The moderator role of parental anxiety
Source: J Health Psychol. 2025 Mar 17;30(13):3998–4012. doi: 10.1177/13591053251323502 (PMC12618725; doi:10.1177/13591053251323502)
Supplement: sj-docx-1-hpq-10.1177_13591053251323502 – Supplemental material for Factors associated with parents’ hesitancy to vaccinate their children against COVID-19: The moderator role of parental anxiety [file sj-docx-1-hpq-10.1177_13591053251323502.docx]

*Descriptive statistics for the key variables*

| Variables | N | M | É.-T. | Min. | Max | |
| --- | --- | --- | --- | --- | --- | --- |
| **Independent Variables** | | | | | |  |
| Mistrust towards Authorities | 439 | 2.35 | 1.40 | 1.00 | 6.00 | |
| Perceived Freedom in Vaccination Decision | 436 | 5.68 | 1.44 | 1.00 | 7.00 | |
| Choice Overload | 434 | 1.99 | 1.31 | 1.00 | 7.00 | |
| Access to Information | 437 | 5.37 | 1.72 | 1.00 | 7.00 | |
| **Moderator** | | | | | |  |
| Parental Anxiety | 438 | 1.89 | 0.45 | 1.00 | 4.00 | |
|  | | | | | |  |
| **Dependent Variable** | | | | | |  |
| Vaccine Hesitancy | 439 | 2.24 | 1.01 | 1.00 | 5.00 | |

*Correlations between variables*

| Variables | 1 | 2 | 3 | 4 | 5 | 6 | 7 | 8 | 9 | 10 | 11 |
| --- | --- | --- | --- | --- | --- | --- | --- | --- | --- | --- | --- |
| 1. Age | --- |  |  |  |  |  |  |  |  |  |  |
| 2. Gender | 0.13** | --- |  |  |  |  |  |  |  |  |  |
| 3. Number of children | -0.30** | -0.10 | --- |  |  |  |  |  |  |  |  |
| 4. Years of education | 0.11* | 0.01 | 0.07 | --- |  |  |  |  |  |  |  |
| 5. Annual family income | 0.24** | 0.15** | 0.00 | 0.37** | --- |  |  |  |  |  |  |
| 6. Mistrust towards authorities | -0.12* | -0.01 | 0.03 | -0.27** | -0.29** | --- |  |  |  |  |  |
| 7. Perceived freedom | 0.03 | -0.04 | -0.03 | 0.11* | 0.14** | -0.39** | --- |  |  |  |  |
| 8. Choice overload | -0.06 | -0.03 | 0.02 | -0.23** | -0.24** | 0.41** | -0.59** | --- |  |  |  |
| 9. Access to information | 0.14** | 0.13** | -0.03 | 0.23** | 0.19** | -0.47** | 0.34** | -0.33** | --- |  |  |
| 10. Parental anxiety | -0.01 | -0.09 | 0.05 | -0.22** | -0.17** | 0.07 | -0.07 | 0.19** | -0.11* | --- |  |
| 11. COVID-19 vaccine hesitancy | -0.16** | -0.02 | 0.03 | -0.33** | -0.33** | 0.83** | -0.39** | 0.42** | -0.48** | 0.05 | --- |

Note. * *p*< 0,05, ** *p*< 0,001. For gender, 1 = female; 2 = male

***Figure 1*.** Parental Anxiety moderates the relationship between Access to Information and Vaccine Hesitancy

***Figure 2*.** Parental Anxiety moderates the relationship between Low Perceived Freedom of Choice and Vaccine Hesitancy
